# Vancomycin and daptomycin dosing recommendations in patients receiving home hemodialysis using Monte Carlo simulation

**DOI:** 10.1186/s12882-023-03314-y

**Published:** 2023-09-14

**Authors:** Susan J. Lewis, Soo Min Jang, Bruce A. Mueller

**Affiliations:** 1 University of Findlay College of Pharmacy, 1000 N. Main Street, Findlay, OH 45840 USA; 2 Mercy Health - St. Anne Hospital, Toledo, OH 43623 USA; 3 Proacture Consulting Group, 6905 Telegraph Rd, Bloomfield Hills, MI 48304 USA; 4 University of Michigan College of Pharmacy, 428 Church Street, Ann Arbor, MI 48109-1065 USA

**Keywords:** Pharmacokinetics, Pharmacodynamics, Vancomycin, Daptomycin, Renal disease, Home hemodialysis, Monte Carlo simulation

## Abstract

**Background:**

Few drug dosing recommendations for patients receiving home hemodialysis (HHD) have been published which has hindered the adoption of HHD. HHD regimens vary widely and differ considerably from conventional, thrice weekly, in-center hemodialysis in terms of treatment frequency, duration and blood and dialysate flow rates. Consequently, vancomycin and daptomycin clearances in HHD are also likely to be different, consequently HHD dosing regimens must be developed to ensure efficacy and minimize toxicity when these antibiotics are used. Many HHD regimens are used clinically, this study modeled ten common HHD regimens and determined optimal vancomycin and daptomycin dosing for each HHD regimen.

**Methods:**

Monte Carlo simulations using pharmacokinetic data derived from the literature and demographic data from a large HHD program treating patients with end stage kidney disease were incorporated into a one-compartment pharmacokinetic model. Virtual vancomycin and daptomycin doses were administered post-HHD and drug exposures were determined in 5,000 virtual patients receiving ten different HHD regimens. Serum concentration monitoring with subsequent dose changes was incorporated into the vancomycin models. Pharmacodynamic target attainment rates were determined for each studied dose. The lowest possible doses that met predefined targets in virtual patients were chosen as optimal doses.

**Results:**

HHD frequency, total dialysate volumes and HHD durations influenced drug exposure and led to different dosing regimens to meet targets. Antibiotic dosing regimens were identified that could meet targets for 3- and 7-h HHD regimens occurring every other day or 4–5 days/week. HHD regimens with 3-day interdialytic periods required higher doses prior to the 3-day period. The addition of vancomycin serum concentration monitoring allowed for calculation of necessary dosing changes which increased the number of virtual subjects meeting pharmacodynamic targets.

**Conclusions:**

Doses of vancomycin and daptomycin that will meet desired pharmacodynamic targets in HHD are dependent on patient and HHD-specific factors. Doses used in conventional thrice weekly hemodialysis are unlikely to meet treatment goals. The antibiotic regimens paired with the HHD parameters studied in this analysis are likely to meet goals but require clinical validation.

**Supplementary Information:**

The online version contains supplementary material available at 10.1186/s12882-023-03314-y.

## Background

Home hemodialysis (HHD) offers a more flexible and convenient dialysis schedule for patients to treat their end stage kidney disease (ESKD), compared to in-center hemodialysis with its rigid thrice-weekly schedule. HHD also allows individualized dialysis delivery with more frequent and/or longer session to meet patient’s solute and fluid control needs. Studies report that HHD is associated with improved survival, cardiovascular outcomes, quality of life, and cost-effectiveness [1–4]. Despite increasing perception of these clinical and lifestyle benefits, HHD is underutilized in the United States. Between 2009 and 2019, the percentage of patients receiving home dialysis (peritoneal dialysis and HHD) only increased from 1.2% to 1.9% [5]. In 2019, only 0.3% of patients who initiate dialysis started with HHD [5].

Recently, the U.S. Department of Health and Human Services launched the Advancing American Kidney Health initiative to improve care for patients with kidney disease and set a goal to significantly increase access and uptake of HHD for ESKD patients [6]. HHD presents an opportunity to reach more ESKD patients to receive dialysis treatment, but several barriers exist preventing its wide utilization. One of them is lack of drug dosing information for HHD patients. HHD regimens vary in frequency, duration and dialysate volume from patient to patient and all differ from thrice-weekly in-center hemodialysis treatments. Thus, optimal dosing of dialyzable drugs for HHD patients may differ from the dosing used in thrice-weekly in-center hemodialysis patients.

Infection remains the second leading cause of morbidity and mortality in ESKD patients [5]. Vancomycin and daptomycin are two most commonly prescribed antibiotic agents to treat methicillin-resistant *Staphylococcus aureus* (MRSA) infections in outpatient dialysis centers [7]. The pharmacokinetics of vancomycin and daptomycin have been well characterized in ESKD patients receiving typical thrice-weekly intermittent hemodialysis (IHD), and both agents are cleared by high-flux dialyzers [8, 9]. No regulatory authorities mandate conducting clinical pharmacokinetic trials of marketed drugs in HHD patients. FDA guidance recommends that pharmacokinetics of drugs be investigated in ESKD patients receiving dialysis, but it primarily refers to thrice-weekly IHD, the most common dialysis modality in the U.S. [10]. Few clinical pharmacokinetic studies of commonly used antibiotics in HHD have been conducted. In lieu of such trials, modeling and simulation can predict the influence of HHD on drug exposure [11, 12] and support clinical dosing decisions. The purpose of the present study was to predict the optimal initial vancomycin and daptomycin dosing regimens in patients receiving common HHD settings using pharmacokinetic modeling and Monte Carlo simulation techniques. Additionally, this study attempted to develop a therapeutic drug monitoring (TDM) strategy to further individualize the subsequent vancomycin regimens in HHD patients because vancomycin serum concentration monitoring is readily available.

## Methods

### Part I. Prediction of optimal vancomycin and daptomycin dosing regimens in patients with HHD

#### Development of pharmacokinetic models

**Fig. 1:**
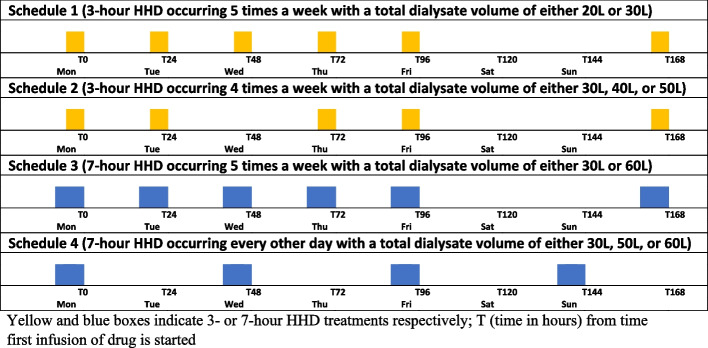
Simulated Home Hemodialysis Schedules

One compartment, first order pharmacokinetic models were developed to construct vancomycin [13, 14] and daptomycin exposure in virtual patients with ESKD receiving HHD. Table 1 outlines the input parameters used in the models. Patients’ body weight data was obtained from a large population of ESKD patients receiving HHD at Fresenius outpatient dialysis centers [internal data] and pharmacokinetic parameters with variances were derived from published vancomycin and daptomycin studies in ESKD patients receiving high-flux dialysis treatments [9, 14–33]. Numerous possible scenarios exist regarding when each HHD session occurs during the week and when to administer drug in relation to HHD session. However, it is not feasible to simulate all possible HHD scenarios. Thus, we devised a fixed HHD schedule for each HHD setting utilizing most frequently used settings in real-world HHD. The models were constructed for 10 different HHD settings with different dialysate flow rates (Qd) and dialysis treatment durations as displayed in Fig. 1. Transmembrane drug clearance in hemodialysis is a function of Qd and saturation coefficient (ratio of dialysate concentration to plasma concentration). Regression analyses were conducted using published data on transmembrane drug clearance and effluent flow rates to estimate saturation coefficients at various Qd in HHD [14, 16, 17, 19, 22–33]. The best fitting relationships were modeled to estimate saturation coefficient at the desired Qd in HHD. The variability of the saturation coefficient expressed as 20% of the standard deviation was incorporated into the models. Body weight was truncated at less than 40 kg with the assumption that virtual patients were adults that weighed at least 40 kg. Patients were assumed to be anuric and therefore had no endogenous renal clearance. All input parameters were assumed to display log-Gaussian distribution. The equations used in the model were as follows:

$${\mathrm{CL}}_{\mathrm{HHD}} =\mathrm{ SA x Qd}$$

$$\mathrm{Ke}\_\mathrm{on }= ({\mathrm{CL}}_{\mathrm{NR}}+{\mathrm{CL}}_{\mathrm{HHD}})/\mathrm{Vd }(\mathrm{intra}-\mathrm{HHD\;period})$$

$$\mathrm{Ke}\_\mathrm{off }= {\mathrm{CL}}_{\mathrm{NR}}/\mathrm{Vd }(\mathrm{inter}-\mathrm{HHD\;period})$$

where CL_HHD_ is the transmembrane clearance during HHD, SA is the saturation coefficient, Qd is the dialysate flow rate, Ke_on is the elimination rate constant during HHD, CL_NR_ is non-renal clearance, Vd is volume of distribution, and Ke_off is the elimination rate constant inter-HHD period.

**Table 1:** Body Weight and Pharmacokinetic Data Used in the Models

	Vancomycin	Daptomycin
Body weight (kg)	93.5 ± 30 [15]	
Vd (L/kg)	0.8 ± 0.24 [14, 16]	0.16 ± 0.04 [9, 17–19]
Non-renal CL (mL/min)	4 ± 1.2 [14, 16]	3.4 ± 1.3 [9, 17]
Unbound fraction of drug (%)	0.82 ± 0.16 [20]	10–12 [21]
Saturation coefficient	0.28-0.49 ± 20% [0–1] [14, 16, 22–30]	0.07-0.12 ± 20% [0–1] [17, 19, 31–33]

#### Pharmacodynamic targets

The vancomycin target for MRSA infection in patients with normal kidney function is the 24-h area under the curve:minimum inhibitory concentration (AUC_24h_:MIC) ratio of 400–600 assuming a MIC of 1 mg/L [34]. This target balances efficacy and the risk of vancomycin-associated nephrotoxicity. However, the upper AUC_24h_ threshold of 600 mg∙h/L associated with nephrotoxicity is of less concern to ESKD patients already receiving dialysis. The simulated HHD patients in this study were assumed to be anuric. Thus, AUC_24h_ ≥ 400 mg∙h/L was used as the primary vancomycin target to find the optimal dose. Additionally, the dose with the mean AUC_24h_ of 400–600 mg∙h/L was preferred to avoid excessive drug exposure and other toxicities.

For daptomycin, the AUC:MIC ratio is also the pharmacodynamic index that is most predictive of bactericidal effect [35, 36], but the precise target of AUC:MIC ratio has not been clearly established. The interquartile ranges (IQR) of AUC_24h_ 465–761 mg∙h/L was suggested as the target efficacy exposure based on the data of patients with severe *S. aureus* infection and CrCl ≥ 30 ml/min, receiving daptomycin 6 mg/kg every 24 h in the largest clinical trial [37, 38]. Additionally, the 75^th^ percentiles of AUC_24h_ 1422 mg∙h/L was proposed as a reasonable safety threshold in another study with individuals with normal renal function who received and well tolerated the highest test dose (12 mg/kg every 24 h) [37, 39]. Thus, we used the IQR of AUC_24h_ 465–1422 mg∙h/L as primary target to determine optimal daptomycin dosing regimens in simulated HHD patients [37].

#### Monte Carlo simulations and prediction of optimal dosing regimen

Various weight-based vancomycin and daptomycin dosing regimens were evaluated in this analysis. All doses were simulated to be infused after HHD ended. Vancomycin infusion time was 1 h for the doses ≤ 10 mg/kg, 2 h for the doses > 10 mg/kg and ≤ 20 mg/kg, and 3 h for the doses > 20 mg/kg. The maximum loading dose (LD) and maintenance dose (MD) for vancomycin were capped as 3 g and 2 g respectively based on the guidelines [34]. Daptomycin infusion time was consistent with 0.5 h for all doses. The maximum LD and MD for daptomycin were capped as 1.5 g [39]. Monte Carlo simulation (MCS)(Crystal Ball Classroom Edition, Oracle) was performed to generate a week of plasma drug concentration–time profiles of 5,000 virtual patients for each tested vancomycin and daptomycin regimen in each of 10 different HHD settings for one week. The AUC_24h_ for each day of vancomycin and daptomycin therapy was computed using the linear trapezoidal rule.

For vancomycin, probability of target attainment (PTA) was calculated by summing up the number of virtual patients attaining the AUC_24h_ ≥ 400 mg∙h/L and dividing by the total number (n = 5,000) of virtual patients. Optimal vancomycin doses were defined as the smallest dose attaining a PTA ≥ 90% preferably with the mean AUC_24h_ 400–600 mg∙h/L in the simulated patients for each HHD setting. For daptomycin, a regimen is considered “optimal” if its resulted IQRs of AUC_24h_ in 5,000 simulated patients was within the target IQR of AUC_24h_ 465–1422 mg∙h/L in each HHD setting.

### Part II. Development of vancomycin therapeutic drug monitoring strategy in patients with HHD

Therapeutic drug monitoring (TDM) is routinely performed for vancomycin therapy. Previously, we developed a technique to integrate TDM and subsequent vancomycin dose individualization into virtual patients receiving different types of renal replacement therapy [11, 40]. Using this technique, we modeled how TDM can be effectively utilized for HHD patients and attempted to develop a practical vancomycin dose adjustment protocol to guide clinicians. In the simulation, TDM in HHD patients used a single pre-dialysis concentration based on the current guideline recommendations [34] and the initial vancomycin doses were adjusted to attain and/or maintain AUC_24h_ of 400–600 mg∙h/L.

The nomogram for dose adjustment protocol was developed based on the predicted vancomycin concentrations in virtual patients with HHD receiving one week of vancomycin dosing regimens recommended from Part I. A single pre-dialysis vancomycin concentration measured immediately prior to the last HHD session of the first week was used as the basis of TDM. The subsequent dose targeting to attain and/or maintain AUC_24h_ of 400–600 mg∙h/L were given after the first HHD of the second week. For example, in a setting where HHD occurs 5 times per week (Mon-Tue-Wed-Thu-Fri), a pre-dialysis concentration was measured prior to HHD session on Friday of the first week of vancomycin therapy, and the adjusted dose was given after the first HHD session of the following week. The decision to adjust the first dose of the next Monday was made for practical reasons. Patients receiving HHD at home would be unlikely to receive TDM results in a timely enough manner to adjust doses any faster. The virtual vancomycin assay results were assumed to be accurate and reflect the model-derived concentrations at that time point. Using the predicted pre-dialysis concentrations and pharmacokinetic profiles selected in the simulation of Part I, the second week of vancomycin concentrations with the adjusted dose was further constructed to estimate AUC_24h_ in each of 5,000 virtual patients. Finally, the equation was derived to individualize a subsequent dose achieving AUC_24h_ of 400–600 mg∙h/L in most patients.

## Results

### Part I. Determination of optimal vancomycin and daptomycin dosing regimens

Tables 2 and 3 display PTA and predicted AUC_24h_ of simulated vancomycin dosing regimens in 10 different HHD settings. MCS analyses indicate that dialysis factors (ie. dialysate flow rate, dialysis treatment duration and interdialytic period) influenced the PTA of simulated vancomycin doses in HHD patients, necessitating different initial dosing regimens in different HHD settings. It is predicted that the vancomycin regimens consisting of a LD of 25 mg/kg post-HHD, followed by MD 5–10 mg/kg administered after each HHD would attain the desired PD target (AUC_24h_ ≥ 400 mg∙h/L) in ~ 90% of simulated patients on each day of the first week of vancomycin therapy with a mean AUC_24h_ of closest to 400–600 mg∙h/L. Notably, for 3-h HHD occurring 4 times per week (Mon-Tue-Thu-Fri) and 7-h HHD occurring 5 times per week (Mon-Tue-Wed-Thu-Fri), a 30–50% higher Friday MD was required to maintain sufficient drug exposure for 3-day interdialytic periods compared to a MD for 1–2-day interdialytic period. Five times per week 3-h HHD occurring (Mon-Tue-Wed-Thu-Fri) with a Qd of 6.7 L/hr or 10 L/hr resulted in lesser dialytic removal (13–17%) during each HHD session, allowing vancomycin administration to occur after HHD only on Mon, Wed and Fri.

**Table 2:** Probability of Target Attainment and Predicted AUC_24h_ of Simulated Vancomycin Dosing Regimens in 3-h Home Hemodialysis Schedule

PTA (%) (percentage of simulated patients attaining AUC _24 h_ < 400 / 400–600 / > 600 mg∙h/L) AUC_24h_ mg∙h/L, Mean ± SD							
Vancomycin Dosing	Day 1 (Mon)	Day 2 (Tue)	Day 3 (Wed)	Day 4 (Thu)	Day 5 (Fri)	Day 6 (Sat)	Day 7 (Sun)
**3-h HHD 5 days per week with a total dialysate volume of 20 L (Qd = 6.7 L/hr)**							
25–5-5–5-7.5 mg/kg post-HHD	96 (4/30/66) 708 ± 218	98 (2/22/76) 749 ± 199	99 (1/20/79) 747 ± 182	99 (1/19/80) 747 ± 173	100 (0/10/90) 831 ± 191	99 (1/18/82) 764 ± 181	98 (2/29/69) 693 ± 170
25–0-5–0-7.5 mg/kg post-HHD^a^	98 (4/29/67) 713 ± 220	92 (8/46/46) 600 ± 159	95 (5/41/54) 703 ± 155	82 (18/63/19) 562 ± 118	97 (3/41/56) 682 ± 148	93 (7/52/41) 628 ± 142	86 (14/60/26) 570 ± 131
**25–0-7.5–0-7.5 mg/kg post-HHD**^a^	**95 (5/29/66)** **712 ± 226**	**91 (9/45/46)** **600 ± 161**	**91 (2/28/70)** **703 ± 177**	**99 (9/56/35)** **562 ± 131**	**96 (1/31/68)** **682 ± 159**	**96 (4/42/54)** **628 ± 150**	**91 (10/53/38)** **570 ± 140**
25–0-10–0-10 mg/kg post-HHD^a^	96 (9/45/46) 712 ± 218	92 (9/45/46) 600 ± 156	99 (1/17/82) 779 ± 192	96 (4/43/53) 623 ± 141	100 (1/12/87) 807 ± 185	99 (1/21/78) 743 ± 175	98 (2/33/65) 674 ± 164
**3-h HHD 5 days per week with a total dialysate volume of 30 L (Qd = 10 L/hr)**							
25–5-5–5-7.5 mg/kg post-HHD	95 (5/28/67) 713 ± 223	98 (2/24/73) 729 ± 192	98 (2/26/72) 709 ± 170	99 (1/28/71) 697 ± 161	100 (0/16/84) 774 ± 178	99 (1/26/73) 712 ± 169	97 (3/40/57) 645 ± 158
25–0-7.5–0-7.5 mg/kg post-HHD^a^	95 (5/29/66) 710 ± 220	90 (10/51/39) 574 ± 145	98 (3/34/63) 665 ± 158	84 (16/63/21) 512 ± 115	97 (3/43/54) 628 ± 140	93 (7/54/39) 579 ± 133	84 (16/59/25) 524 ± 125
**25–0-10–0-10 mg/kg post-HHD**^a^	**96 (4/30/66)** **713 ± 222**	**90 (10/51/39)** **575 ± 146**	**99 (1/22/77)** **741 ± 180**	**93 (7/56/37)** **570 ± 130**	**100 (0/19/81)** **750 ± 171**	**99 (2/29/69)** **691 ± 162**	**96 (4/43/53)** **625 ± 151**
**3-h HHD 4 days per week with a total dialysate volume of 30 L (Qd = 10 L/hr)**							
20–5-5–5 mg/kg post-HHD	88 (12/45/43) 592 ± 179	94 (6/41/53) 627 ± 163	89 (11/52/37) 570 ± 148	93 (7/51/42) 586 ± 141	96 (4/48/48) 609 ± 142	91 (9/57/34) 560 ± 134	81 (19/60/21) 507 ± 125
20–5-5–7.5 mg/kg post- HHD	88 (12/45/43) 590 ± 175	94 (5/41/53) 625 ± 161	89 (11/51/28) 569 ± 147	93 (7/50/43) 585 ± 140	98 (2/30/68) 685 ± 161	96 (4/42/54) 630 ± 152	90 (10/52/38) 571 ± 142
25–5-5–5 mg/kg post- HHD	95 (5/29/66) 714 ± 223	98 (2/24/74) 737 ± 194	96 (4/33/63) 670 ± 177	97 (3/35/62) 663 ± 159	98 (2/33/65) 669 ± 154	95 (5/46/49) 615 ± 146	89 (11/55/34) 557 ± 136
**25–5-5–7.5 mg/kg** **post-HHD**	**96 (4/30/66)** **709 ± 223**	**98 (2/24/74)** **732 ± 192**	**96 (4/35/61)** **664 ± 175**	**97 (3/36/61)** **657 ± 157**	**99 (1/20/79)** **740 ± 171**	**98 (2/32/66)** **680 ± 162**	**95 (5/45/50)** **616 ± 151**
**3-h HHD 4 days per week with a total dialysate volume of 40 L (Qd = 13.3 L/hr)**							
20–5-5–5 mg/kg post- HHD	89 (12/46/42) 591 ± 174	94 (6/45/49) 612 ± 152	88 (12/55/33) 555 ± 139	91 (9/56/35) 562 ± 131	94 (6/55/39) 578 ± 131	87 (13/61/26) 532 ± 124	75 (25/61/14) 481 ± 116
20–5-5–7.5 mg/kg post- HHD	87 (13/46/41) 585 ± 174	93 (7/46/47) 606 ± 154	87 (13/54/33) 551 ± 141	90 (10/56/34) 558 ± 133	98 (2/38/60) 650 ± 152	94 (6/48/46) 600 ± 145	86 (14/56/30) 542 ± 135
25–5-5–5 mg/kg post- HHD	95 (5/29/66) 712 ± 222	98 (2/26/72) 717 ± 184	96 (4/37/59) 650 ± 167	97 (3/42/55) 631 ± 148	97 (3/43/54) 630 ± 144	93 (7/53/40) 580 ± 136	84 (16/59/25) 525 ± 127
**25–5-5–7.5 mg/kg** **post-HHD**	**95 (5/30/65)** **704 ± 218**	**98 (2/27/71)** **709 ± 181**	**96 (5/39/57)** **643 ± 165**	**97 (4/44/53)** **625 ± 146**	**99 (1/28/71)** **700 ± 159**	**97 (3/40/57)** **644 ± 151**	**93 (7/52/41)** **582 ± 141**
**3-h HHD 4 days per week with a total dialysate volume of 50 L (Qd = 16.7 L/hr)**							
20–5-5–5 mg/kg post- HHD	88 (12/46/42) 587 ± 176	93 (7/47/46) 598 ± 149	86 (14/56/30) 542 ± 135	89 (11/59/30) 544 ± 126	91 (9/57/34) 557 ± 127	83 (17/62/21) 512 ± 120	69 (31/58/11) 463 ± 112
20–5-5–7.5 mg/kg post- HHD	88 (12/45/43) 590 ± 176	93 (7/47/46) 601 ± 151	86 (14/55/31) 545 ± 138	89 (11/59/30) 546 ± 129	97 (3/51/42) 636 ± 148	93 (7/51/42) 586 ± 140	85 (15/59/26) 529 ± 130
25–5-5–5 mg/kg post- HHD	95 (5/30/65) 703 ± 219	97 (3/28/69) 698 ± 178	95 (5/40/55) 633 ± 162	95 (5/46/49) 609 ± 143	95 (5/48/47) 604 ± 139	90 (10/57/33) 556 ± 131	79 (21/60/19) 502 ± 123
**25–5-5–7.5 mg/kg** **post-HHD**	**96 (4/30/66)** **707 ± 216**	**98 (2/28/70)** **703 ± 176**	**96 (4/40/56)** **638 ± 161**	**96 (4/46/50)** **614 ± 143**	**99 (1/30/69)** **687 ± 157**	**97 (3/42/55)** **633 ± 149**	**91 (9/54/37)** **571 ± 139**

*Qd* Dialysate flow rate, *PTA* Probability of target attainment

^a^Vancomycin is given only on day 1, 3 and 5 only for these drug regimens

**Bolded dosing regimens are optimal doses attaining PTA ≥ 90% with a mean AUC**_**24h**_** closest to 400–600 mg∙h/L**

**Table 3:** Probability of Target Attainment and Predicted AUC_24h_ of Simulated Vancomycin Dosing Regimens in 7-h Home Hemodialysis Schedule

PTA (%) (percentage of simulated patients attaining AUC _24 h_ < 400 / 400–600 / > 600 mg∙h/L) AUC_24h_ mg∙h/L, Mean ± SD							
Vancomycin Dosing	Day 1 (Mon)	Day 2 (Tue)	Day 3 (Wed)	Day 4 (Thu)	Day 5 (Fri)	Day 6 (Sat)	Day 7 (Sun)
**7-h HHD 5 days per week with a total dialysate volume of 30 L (Qd = 4.3 L/hr)**							
25–5-5–5-5 mg/kg post-HHD	95 (5/31/64) 693 ± 210	97 (3/32/65) 673 ± 164	97 (3/56/41) 633 ± 142	96 (4/48/48) 608 ± 135	96 (4/48/48) 610 ± 137	91 (9/57/34) 561 ± 129	79 (21/60/19) 498 ± 119
**25–5-5–5-7.5 mg/kg post-HHD**	**95 (5/32/63)** **686 ± 204**	**97 (3/33/64)** **669 ± 162**	**97 (3/42/55)** **630 ± 142**	**96 (4/48/48)** **606 ± 136**	**99 (1/31/68)** **684 ± 153**	**97 (3/42/55)** **630 ± 145**	**90 (10/56/34)** **559 ± 133**
25–5-5–5-10 mg/kg post-HHD	95 (5/32/63) 691 ± 210	97 (3/32/65) 673 ± 165	97 (3/41/56) 633 ± 143	96 (4/46/50) 609 ± 136	100 (0/16/84) 765 ± 170	99 (1/26/73) 706 ± 161	96 (4/42/54) 626 ± 147
25–7.5–7.5–7.5–10 mg/kg post-HHD	95 (5/32/63) 688 ± 211	99 (1/21/78) 743 ± 185	100 (0/18/82) 757 ± 173	100 (0/16/84) 771 ± 173	100 (0/5/95) 885 ± 199	100 (0/12/88) 815 ± 189	99 (1/24/75) 723 ± 173
**7-h HHD 5 days per week with a total dialysate volume of 60 L (Qd = 8.6 L/hr)**							
25–5-5–5-5 mg/kg post-HHD	94 (6/33/61) 680 ± 203	95 (5/49/46) 599 ± 136	88 (12/62/26) 530 ± 118	78 (22/62/16) 490 ± 114	93 (7/57/36) 565 ± 126	86 (14/62/24) 522 ± 119	67 (33/57/10) 456 ± 109
25–5-5–5-7.5 mg/kg post-HHD	95 (5/33/62) 676 ± 200	98 (2/33/65) 668 ± 152	98 (2/38/60) 646 ± 140	97 (3/40/57) 636 ± 141	98 (2/35/63) 662 ± 148	96 (4/46/50) 610 ± 140	86 (14/58/28) 534 ± 129
**25–5-5–5-10 mg/kg post-HHD**	**95 (5/32/63)** **683 ± 200**	**98 (2/31/67)** **675 ± 152**	**98 (2/37/61)** **652 ± 141**	**98 (2/40/58)** **642 ± 142**	**100 (0/19/81)** **745 ± 162**	**99 (1/30/69)** **688 ± 153**	**95 (5/49/46)** **601 ± 140**
**7-h HHD every other day with a total dialysate volume of 30 L (Qd = 4.3 L/hr)**							
20 mg/kg LD, 7.5 mg/kg post-HD	89 (11/46/43) 596 ± 182	83 (17/50/33) 550 ± 163	95 (5/42/53) 633 ± 164	89 (11/53/36) 565 ± 145	97 (3/39/58) 648 ± 157	92 (8/52/40) 579 ± 142	98 (2/36/62) 661 ± 158
25 mg/kg LD, 5 mg/kg post-HD	95 (5/28/67) 716 ± 220	94 (6/33/61) 676 ± 202	96 (4/38/58) 649 ± 162	91 (9/51/40) 577 ± 143	93 (7/52/41) 581 ± 134	84 (16/61/23) 518 ± 122	89 (11/61/28) 540 ± 124
**25 mg/kg LD, 7.5 mg/kg post-HHD**	**96 (4/28/68)** **717 ± 219**	**95 (5/33/62)** **677 ± 200**	**98 (2/24/74)** **725 ± 181**	**96 (4/37/59)** **646 ± 160**	**99 (1/26/73)** **707 ± 164**	**96 (4/41/55)** **630 ± 148**	**99 (1/27/72)** **698 ± 160**
**7-h HHD every other day with a total dialysate volume of 50 L (Qd = 7.1 L/hr)**							
20 mg/kg LD, 7.5 mg/kg post-HHD	89 (11/45/46) 602 ± 182	84 (16/51/33) 549 ± 159	94 (6/48/46) 601 ± 145	86 (14/60/26) 530 ± 127	95 (5/51/44) 594 ± 135	86 (14/61/25) 525 ± 122	95 (5/52/43) 594 ± 134
25 mg/kg LD, 5 mg/kg post-HHD	96 (4/28/68) 718 ± 225	94 (6/34/60) 670 ± 200	95 (5/48/47) 603 ± 142	86 (14/60/26) 530 ± 124	85 (15/64/21) 516 ± 114	68 (32/59/9) 456 ± 104	73 (27/62/11) 469 ± 105
**25 mg/kg LD, 7.5 mg/kg post-HHD**	**95 (5/28/67)** **719 ± 228**	**94 (6/34/60)** **671 ± 204**	**98 (2/32/66)** **681 ± 167**	**94 (6/48/46)** **600 ± 146**	**97 (3/39/58)** **641 ± 147**	**91 (9/55/36)** **566 ± 132**	**96 (4/44/52)** **621 ± 142**
**7-h HHD every other day with a total dialysate volume of 60 L (Qd = 8.6 L/hr)**							
20 mg/kg LD, 7.5 mg/kg post-HHD	89 (11/45/44) 598 ± 179	83 (17/51/32) 543 ± 155	93 (7/52/41) 583 ± 139	83 (17/61/22) 512 ± 122	93 (7/57/36) 569 ± 130	81 (19/62/19) 501 ± 117	92 (8/57/35) 565 ± 129
25 mg/kg LD, 5 mg/kg post-HHD	95 (5/29/66) 714 ± 225	94 (6/36/58) 663 ± 199	93 (7/52/41) 584 ± 138	83 (17/61/22) 512 ± 121	79 (21/63/16) 493 ± 112	60 (40/53/7) 434 ± 102	64 (36/56/8) 445 ± 103
**25 mg/kg LD, 7.5 mg/kg post-HHD**	**95 (5/28/67)** **714 ± 226**	**93 (7/34/59)** **664 ± 200**	**97 (3/34/63)** **660 ± 156**	**92 (8/52/40)** **580 ± 136**	**96 (4/46/50)** **614 ± 136**	**89 (11/60/29)** **541 ± 122**	**95 (5/51/44)** **592 ± 131**
25 mg/kg LD, 10 mg/kg post-HHD	96 (4/29/67) 717 ± 222	94 (6/35/59) 666 ± 196	99 (1.21/78) 739 ± 176	97 (3/38/59) 650 ± 153	99 (1/20/79) 737 ± 165	98 (2/38/60) 649 ± 148	99 (1/19/80) 741 ± 165

*Qd* dialysate flow rate, *PTA* probability of target attainment, *LD* loading dose

Bolded dosing regimens are optimal doses attaining PTA ≥ 90% with a mean AUC_24h_ closest to 400–600 mg∙h/L

**Fig. 2:**
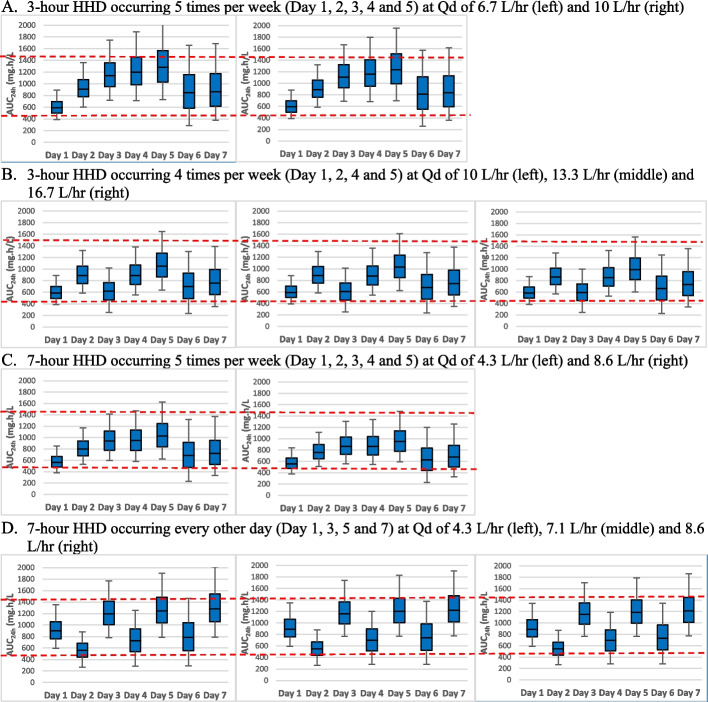
One Week of Predicted AUC_24h_ IQR with Model-recommended Daptomycin Regimens in Ten Home Hemodialysis Settings

One week of predicted AUC_24h_ IQRs of simulated daptomycin dosing regimens in 10 different HHD settings are reported in the supplementary material. MCS results indicated that daptomycin 4 mg/kg post-HHD with a 2 mg/kg supplemental dose on the 3^rd^ day of 3-day interdialytic period would be optimal in asymmetrical HHD settings where HHD occurs 4–5 times per week. Daptomycin dosing regimens without a supplementary dose on the last day of 3-day interdialytic period did not meet the target IQR range of AUC_24h_ (465–1422 mg∙h/L) during each day of 3-day interdialytic period. For symmetrical HHD settings where HHD occurs every other day, daptomycin 6 mg/kg given after HHD would be optimal, attaining the target IQR of AUC24h. Figure 2 depicts one week of the predicted IQR of AUC_24h_ with our model-recommended daptomycin dosing regimens in ten different HHD settings.

### Part II. Vancomycin therapeutic drug monitoring strategy in patients with HHD

MCS results suggested that TDM targeting a pre-dialysis concentration of 24 mg/L would ensure AUC_24h_ ≥ 400 mg∙h/L in ≥ 90% of virtual patients in all HHD settings, following the model-recommended initial doses for a week. Thus, the new MD was to be proportionally adjusted from the initial MD to attain a pre-dialysis concentration of 24 mg/L as the equation below. Of note, in HHD settings where a HHD occurs 4–5 times per week (Mon-Tue-Thu-Fri or Mon-Tue-Wed-Thu-Fri), a 30% higher Friday dose was necessary to maintain PTA ≥ 90% on the third day of a 3-day interdialytic period.

$$\mathrm{New\;vancomycin\;maintenance\;dose}=\frac{\text{Previous maintenance dose x 24 mg/L }}{\text{Pre-dialysis vancomycin concentration}}$$

**Fig. 3:**
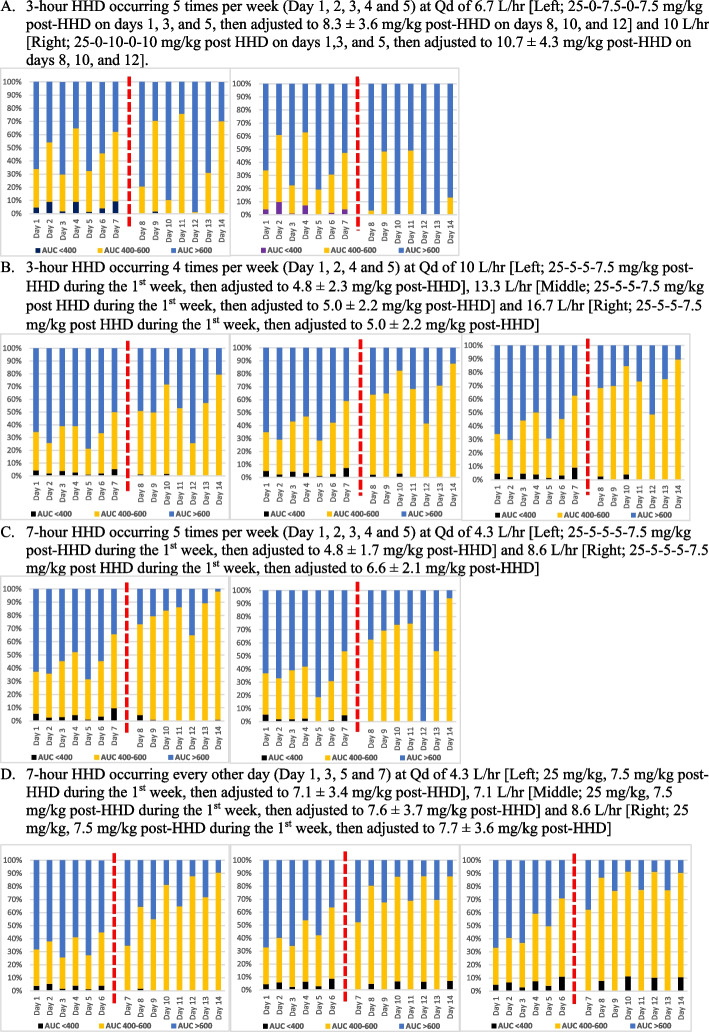
Frequency of Mean Vancomycin AUC_24h_ Before and After Virtual TDM

Figure 3A-D illustrate the proportions of simulated patients (*n* = 5,000) attaining AUC_24h_ < 400, 400–600, and ≥ 600 mg∙h/L during 2 weeks of vancomycin therapy (ie. initial recommended regimens for the first week, followed by subsequently adjusted MD directed by TDM for the second week). Overall, the TDM-guided individualized dosing strategy with a target pre-dialysis concentration of 24 mg/L yielded a higher proportion of patients attaining AUC_24h_ 400–600 mg∙h/L, and decreased the proportions of those with sub-therapeutic (AUC_24h_ < 400 mg∙h/L) or excessive drug exposure (AUC_24h_ > 600 mg∙h/L), compared to those resulted from the initial regimens. The mean AUC_24h_ on each day after dose adjustment with TDM ranged from 400 to 600 mg∙h/L, with an exception of 3-h HHDs occurring 5 times per week (Mon-Tue-Wed-Thu-Fri). In this setting, the recommended dosing regimen needed to be given less frequently (i.e. Mon-Wed-Fri only), but a higher MD was necessary to ensure the target attainment on days when vancomycin was not given.

## Discussion

This is the first in silico study to determine the optimal initial dosing recommendations of vancomycin and daptomycin in patients receiving HHD in various dialysis regimens. The MCS techniques enabled us to assess the PTA of various vancomycin and daptomycin dosing regimens in each of ten different HHD settings and to predict the ones that are likely to attain the therapeutic targets in most patients. As expected, optimal vancomycin and daptomycin dosing regimens for HHD (Table 4) would differ from usual doses recommended for thrice weekly high-flux IHD (25 mg/kg LD, 10 mg/kg MD post-dialysis for vancomycin and 4–6 mg/kg post-dialysis for daptomycin) [21, 34].

**Table 4:** Optimal Vancomycin and Daptomycin Dosing Regimens in Ten Home Hemodialysis settings

HHD Regimen			Vancomycin Dosing Regimen		Daptomycin Dosing Regimen
**Duration** **(Hours/** **session)**	**Frequency** **(Days/week)**	**Dialysate Volume (L/session)**	**Initial** **Dosing Regimen**	**TDM strategy after the initial dosing regimen**	
3	5 (M-T-W-Th-F)	20	25–0-7.5–0-7.5 mg/kg post-HHD^a^	1) Draw pre-HHD level prior to the last HHD of the week (i.e. Friday) 2) Adjust the dose using the equation below: $\text{New MD (mg/kg) = }\frac{\text{Previous MD x 24 mg/L}}{\text{Pre-HHD vancomycin conc.}}$	4 mg/kg post-HHD, with 2 mg/kg supplemental dose on the 3^rd^ day of 3-day interdialytic period
		30	25–0-10–0-10 mg/kg post-HHD^a^		
3^¶^	4 (M-T-Th-F)	30	25–5-5–7.5 mg/kg post-HHD		
		40			
		50			
7^¶^	5 (M-T-W-Th-F)	30	25–5-5–5-7.5 mg/kg post-HHD		
		60	25–7.5–7.5–7.5–10 mg/kg post-HHD		
7	3.5 (M-W–F-Sun)	30	25 mg/kg, 7.5 mg/kg post-HHD		6 mg/kg post-HHD
		50			
		60			

*HHD* home hemodialysis, *TDM* Therapeutic drug monitoring, *MD* Maintenance dose

^a^Doses given on M-W–F only; ^¶^For these HHD settings, add 30% to the newly calculated MD for any 3-day interdialytic period

For vancomycin, HHD variables including the frequency, duration and dialysate flow rates influenced vancomycin exposure (AUC_24h_) thereby altering the optimal dosing regimen with different HHD regimens, as shown in Tables 2 and 3. For example, 7-h HHD occurring 5 times a week (Mon-Tue-Wed-Thu-Fri) with Qd of 4.3 L/hr required 25 mg/kg LD, 5–5-5–7.5 mg/kg post-HHD to attain the PD target on each day for one week, while the same HHD with Qd of 8.6 L/hr necessitated 25 mg/kg LD, 5–5-5–10 mg/kg dosing to meet targets. The optimal vancomycin doses for 7-h HHD with Qd 4.3 L/hr was 25 mg/kg LD, 7.5 mg/kg post-HHD if occurring every other day, but 25 mg/kg LD, 5–5-5–7.5 mg/kg would be optimal if the same HHD session occurred 5 times a week (Mon-Tue-Wed-Thu-Fri), highlighting the impact of HHD frequency on required dosing regimens. In addition to efficacy/safety targets, practical dosing considerations were explored via MCS. For 3-h HHD with Qd 6.7 L/hr occurring 5 times a week, we determined that 25 mg/kg LD, 0–7.5–0-7.5 mg/kg post-HHD (ie. no dose given on Tue and Thur) would be better than 25 mg/kg LD, 5–5-5–7.5 mg/kg post-HHD (ie. 5–7.5 mg/kg given after each of all HHD sessions) because it reduced the dosing frequency with smaller total weekly doses while attaining the efficacy target with lower AUC_24h_ on each day compared to the latter regimen.

This present study also used the previously developed “virtual TDM” technique [11, 40] to mimic the clinical situation and further guide the subsequent dosing for clinicians. The MCS analysis indicated that targeting pre-dialysis concentrations of 20–24 mg/L after one week of our recommended initial regimens would attain and/maintain an AUC_24h_ ≥ 400 mg∙h/L for the following week in most simulated patients with 10 different HHD (Fig. 3). Thus, virtual TDM was designed to target a pre-dialysis concentration of 24 mg/L to ensure the target attainment in virtual patients with all simulated HHD settings. This pre-dialysis concentration target may result in a slightly higher new MD than necessary in some HHD settings. For example, the adjusted MD after TDM using this target pre-dialysis concentration in 3-h HHD occurring 5 times a week resulted in the target (AUC_24h_ ≥ 400 mg∙h/L) attainment in almost all virtual patients, but a higher proportion of patients had AUC_24h_ ≥ 600 mg∙h/L. However, this pre-dialysis concentration of 24 mg/L was found to be the best predictor for attaining targets in all simulated HHD settings. This target pre-dialysis concentration of 24 mg/L is also higher than those (15–20 mg/L) recommended in patients receiving thrice weekly IHD in the vancomycin consensus guidelines [33]. Of note, this guideline recommendation is based on another MCS study showing that pre-dialysis vancomycin concentrations of 10–20 mg/L would results in mean AUC_24h_ from 250 to 450 mg·h/L in patients receiving thrice IHD [34, 40]. The mean new TDM-based vancomycin MD in all HHD settings were not significantly different from the model-recommended initial MD in each HHD setting, but some virtual patients are predicted to need a new MD that was different from the initial dose to attain the target (Fig. 3). Based on these findings, we recommend weekly TDM to ensure all HHD patients to receive the optimal doses during prolonged vancomycin therapy. Any change in patient’s clinical condition and/or HHD modification also warrant TDM to ensure target attainment in these patients.

Optimal daptomycin dosing regimens (Table 4) differed whether HHD occurs symmetrically (ie. every other day) or asymmetrically (ie. 4–5 times a week). MCS predicted that daptomycin 6 mg/kg post-HHD would be optimal in simulated symmetrical HHD settings, but would not successfully attain the target range (IQR range of AUC_24h_ 465–1422 mg∙h/L) in asymmetrical HHD settings. In asymmetrical HHD settings, modeled HHD had a 3-day interdialytic period (ie. Fri-Mon). Simulated fixed dosing regimens (4–8 mg/kg post-HHD) with or without a higher dose on the first day (ie. Fri) of 3-day interdialytic period did not fall within the target IQR range of AUC_24h_._._ For example, in 3-h HHD with Qd 10 L/hr occurring 4 times a week (Mon-Tue-Thu-Fri), daptomycin 4 mg/kg post-HHD for a 3-day interdialytic period (Fri-Sun) attained the target IQR range of AUC_24h_ during the first two days (ie. Fri and Sat), but was not sufficient to maintain the target on the last day (ie. Sun) with an AUC_24h_ IQR range of 257–698 mg∙h/L. To meet the challenge of attaining the target on the Sunday, we simulated 4 mg/kg post-HHD with a 6 mg/kg post-HHD prior to the 3-day interdialytic period in the same HHD setting. Although daptomycin 6 mg/kg post-HHD on day 5 (ie. Fri) achieved the IQR AUC_24h_ target range on the last day of 3-day interdialytic period (ie. Sun), this dose resulted in the IQR AUC_24h_ range of 1146–1640 mg∙h/L on the first day (Fri) of 3-day interdialytic period, exceeding the target IQR range (465–1422 mg∙h/L). Thus, daptomycin dosing regimen utilizing a supplementary dose on the last day of 3-day interdialytic period was simulated to attain the target range during each day of 3-day interdialytic period. MCS showed that daptomycin 4 mg/kg post-HHD with a 2 mg/kg supplementary dose on 3rd day of 3-day interdialytic period would be optimal in all asymmetric HHD settings.

This study has several limitations to consider before clinicians apply the findings from the MCS analysis in practice. First, pharmacokinetic modeling and simulations were performed based on the assumption that patients are > 40 kg adults receiving HHD and were anuric. It was also assumed that no changes in pharmacokinetic parameters and HHD regimen occurred during the modeled period. Virtual patients were constructed based on demographic information from ESKD patients receiving HHD and pharmacokinetic characteristics with variances from the published studies conducted in ESKD patients receiving dialysis. It should be noted that we did not model all possible HHD scenarios, but only in ten common HHD Qd and treatment durations within a fixed schedule where antibiotic doses are initiated on Monday after HHD. Hence, our model-recommended doses should be applied to only anuric patients with similar demographic characteristics and HHD scenarios. If vancomycin therapy is initialed on Friday, we would recommend a 30% higher LD with a maximum dose of 3,000 mg [34] for a 3-day interdialytic period. The recommended initial MD can be given for the following week and TDM can be performed on Friday of that same following week to determine the subsequent MD attaining the target for an individual patient. In contrast, daptomycin therapy started on Friday in patients receiving asymmetric HHD, 4 mg/kg dose can be given after HHD on Friday with a supplemental dose of 2 mg/kg on Sunday. Thereafter our model-recommended daptomycin doses can be followed for the following week. Secondly, we modeled vancomycin infusion rates to be 1 h for the doses ≤ 10 mg/kg, 2 h for the doses > 10 mg/kg and ≤ 20 mg/kg, and 3 h for the doses > 20 mg/kg with max doses of 3 g and 2 g for LD and MD respectively. This would result in faster infusion rates than typical practice (e.g. 1 g over 1 h or 2 g over 2 h) in larger virtual patients (ie. body weight > 100 kg). However, such standardization was necessary to simulate each vancomycin dosing in 5,000 patients simultaneously. Third, we primarily used the “efficacy” target (AUC_24h_ ≥ 400 mg∙h/L assuming the MRSA MIC of 1 mg/L) to select initial vancomycin dosing recommendation and the target pre-dialysis concentration for TDM. However, these selected doses and the target pre-dialysis concentration resulted in high drug exposure exceeding the toxicity threshold (AUC_24h_ ≥ 600 mg∙h/L) in some virtual patients. Vancomycin-induced nephrotoxicity may be less of concern, but potentially increased risk of other vancomycin toxicity including ototoxicity should be noted. Thus, prior to the application of our model-recommended initial vancomycin dose, we recommend clinicians consider patient’s body weight, HHD setting and clinical status to weigh the benefit vs. toxicity risk. TDM should be performed to further optimize individual patient’s subsequent vancomycin dose. Finally, daptomycin dosing regimens were simulated only for 1 week to determine optimal dose in each of ten HHD settings in this present study. However, HHD patients may receive more than one week of daptomycin therapy. As patients continue our recommended initial daptomycin doses, drug accumulation may occur in some patients. Unfortunately, TDM with daptomycin therapy is not commonly available. Thus, we strongly recommend clinicians monitor CPK at least once a week to assess the increased risk of muscle toxicity, as well as the related symptoms such as myopathy or weakness of the extremities.

## Conclusion

As the use of HHD grows, vancomycin and daptomycin will be used increasingly to treat gram-positive infections. While clinical validation of our findings is necessary, this MCS suggests that the variety of HHD regimens used in clinical practice affects vancomycin and daptomycin doses required to achieve therapeutic target. We were able to develop initial vancomycin and daptomycin dosing regimens and vancomycin TDM strategies for ten of the most used HHD regimens.

### Supplementary Information

**Additional file 1: Table 1 **Predicted AUC_24h_ interquartile range of daptomycin dosing regimens in patients receiving 3-hour home hemodialysis. **Table 2** Predicted AUC_24h_ interquartile range of daptomycin dosing regimens in patients receiving 7-hour home hemodialysis. 

## Data Availability

The datasets used and/or analyzed during the current study are available from the corresponding author on reasonable request.

## Abbreviations

AUC_24h_: 24-Hour area under the curve
MIC: Minimum inhibitory concentration
ESKD: End stage kidney disease
HHD: Home hemodialysis
IHD: Intermittent hemodialysis
IQR: Interquartile ranges
LD: Loading dose
MCS: Monte Carlo simulation
MD: Maintenance dose
Qd: Dialysate flow rate
MRSA: Methicillin-resistant *Staphylococcus aureus*
PTA: Probability of target attainment
TDM: Therapeutic drug monitoring
