# Supplementary material for: Vancomycin and daptomycin dosing recommendations in patients receiving home hemodialysis using Monte Carlo simulation
Source: BMC Nephrol. 2023 Sep 14;24:270. doi: 10.1186/s12882-023-03314-y (PMC10500909; doi:10.1186/s12882-023-03314-y)
Supplement: Supplementary file 1 — Additional file 1: Table 1 Predicted AUC24h interquartile range of daptomycin dosing regimens in patients receiving 3-hour home hemodialysis. Table 2 Predicted AUC24h interquartile range of daptomycin dosing regimens in patients receiving 7-hour home hemodialysis. [file 12882_2023_3314_MOESM1_ESM.pdf]

Supplementary material

Table 1. Predicted AUC_{24h} Interquartile Range of Daptomycin Dosing Regimens in Patients Receiving 3-hour Home Hemodialysis

Interquartile range of AUC_{24h} (mg·h/L) in 5000 simulated HHD patients							
Daptomycin Dosing	Day 1 (Mon)	Day 2 (Tue)	Day 3 (Wed)	Day 4 (Thu)	Day 5 (Fri)	Day 6 (Sat)	Day 7 (Sun)
**3-hour HHD 5 days per week with a total dialysate volume of 20 L (Qd = 6.7 L/hr)**							
4 mg/kg post-HHD	497-701	768-1067	941-1345	970-1439	1018-1552	562-1146	298-849
**4 mg/kg post-HHD with 2 mg/kg SD on day 7**	**501-699**	**776-1073**	**952-1359**	**983-1455**	**1030-1568**	**582-1160**	**615-1172**
4-4-4-4-6 mg/kg post-HHD	501-697	769-1066	942-1344	971-1441	1287-1883	723-1375	386-1017
4-4-4-4-8 mg/kg post-HHD	496-696	762-1068	939-1345	971-1433	1558-2211	874-1601	467-1179
6 mg/kg post-HHD	749-1052	1153-1607	1419-2036	1461-2174	1523-2339	848-1731	448-1280
**3-hour HHD 5 days per week with a total dialysate volume of 30 L (Qd = 10 L/hr)**							
4 mg/kg post-HHD	502-693	767-1052	928-1316	954-1399	995-1501	555-1104	294-818
**4 mg/kg post-HHD with 2 mg/kg SD on day 7**	**496-696**	**758-1057**	**924-1324**	**949-1408**	**989-1510**	**550-1113**	**591-1130**
4-4-4-4-6 mg/kg post-HHD	497-704	758-1063	920-1334	944-1414	1258-1855	704-1363	377-1005
4-4-4-4-8 mg/kg post-HHD	499-705	764-1059	935-1325	961-1407	1546-2182	883-1584	475-1168
6 mg/kg post-HHD	749-1050	1135-1583	1385-1986	1427-2113	1486-2268	823-1661	434-1212
**3-hour HHD 4 days per week with a total dialysate volume of 30 L (Qd = 10 L/hr)**							
4 mg/kg post-HHD	501-698	762-1051	465-775	742-1081	873-1291	486-944	257-698
**4 mg/kg post-HHD with 2 mg/kg SD on day 7**	**494-699**	**749-1051**	**469-767**	**737-1070**	**865-1272**	**490-930**	**560-994**
4-4-4-4-6 mg/kg post-HHD	499-698	760-1063	466-782	744-1088	1146-1640	648-1187	345-871
4-4-4-4-8 mg/kg post-HHD	497-697	760-1062	466-780	742-1087	1407-1974	806-1418	433-1045
6 mg/kg post-HHD	745-1042	1137-1578	696-1153	1107-1614	1305-1027	727-1408	383-1037
**3-hour HHD 4 days per week with a total dialysate volume of 40 L (Qd = 13.3 L/hr)**							
4 mg/kg post-HHD	500-695	747-1032	452-757	720-1047	841-1237	468-905	246-667
**4 mg/kg post-HHD with 2 mg/kg SD on day 7**	**501-701**	**751-1039**	**456-757**	**721-1049**	**844-1236**	**475-903**	**548-975**
4-4-4-4-6 mg/kg post-HHD	499-697	747-1046	457-765	722-1060	1110-1595	637-1145	346-836
4-4-4-4-8 mg/kg post-HHD	497-696	750-1040	462-763	728-1058	1387-1945	802-1391	432-1014
6 mg/kg post-HHD	745-1043	1119-1551	684-1131	1080-1571	1261-1848	709-1349	376-986
**3-hour HHD 4 days per week with a total dialysate volume of 50 L (Qd = 16.7 L/hr)**							
4 mg/kg post-HHD	495-696	726-1022	445-737	701-1015	808-1182	454-867	238-632
**4 mg/kg post-HHD with 2 mg/kg SD on day 7**	**495-692**	**729-1021**	**449-744**	**705-1024**	**815-1196**	**464-879**	**540-958**
4-4-4-4-6 mg/kg post-HHD	493-698	727-1017	444-743	704-1019	1082-1533	615-1099	331-798
4-4-4-4-8 mg/kg	493-693	731-1018	450-745	708-1022	1349-1877	782-1343	418-979

post-HHD							
6 mg/kg post-HHD	744-1035	1101-1519	663-1113	1055-1529	1220-1787	679-1315	354-966

Qd: dialysate flow rate; SD: supplemental dose

Shaded boxes indicate the day of a 3-hour HHD session.

Bolded dosing regimens are those with the predicted AUC_{24h} IQRs of 5,000 simulated patients falling within the target AUC_{24h} IQR 465-1422 mg·h/L

Table 2. Predicted AUC_{24h} Interquartile Range of Daptomycin Dosing Regimens in Patients Receiving 7-hour Home Hemodialysis

Interquartile range of AUC_{24h} (mg·h/L) in 5000 simulated HHD patients							
Daptomycin Dosing	Day 1 (Mon)	Day 2 (Tue)	Day 3 (Wed)	Day 4 (Thu)	Day 5 (Fri)	Day 6 (Sat)	Day 7 (Sun)
**7-hour HHD 5 days per week with a total dialysate volume of 30 L (Qd = 4.3 L/hr)**							
4 mg/kg post-HHD	482-671	684-949	787-1130	782-1155	844-1263	475-930	244-667
**4 mg/kg post-HHD with 2 mg/kg SD on day 7**	**483-666**	**680-939**	**777-1113**	**774-1134**	**838-1245**	**471-915**	**526-951**
4-4-4-4-6 mg/kg post-HHD	479-665	672-938	775-1112	771-1144	1107-1586	619-1148	317-818
4-4-4-4-8 mg/kg post-HHD	480-664	675-940	773-1121	771-1151	1371-1933	785-1398	401-984
6 mg/kg post-HHD	718-994	1015-1403	1165-1676	1154-1718	1249-1874	694-1372	354-978
**7-hour HHD 5 days per week with a total dialysate volume of 60 L (Qd = 8.6 L/hr)**							
4 mg/kg post-HHD	472-656	645-889	722-1027	712-1040	778-1146	438-842	220-592
**4 mg/kg post-HHD with 2 mg/kg SD on day 7**	**473-659**	**648-895**	**724-1027**	**713-1039**	**779-1141**	**440-833**	**500-884**
4-4-4-4-6 mg/kg post-HHD	476-655	650-890	729-1025	719-1037	1061-1480	596-1071	300-744
4-4-4-4-8 mg/kg post-HHD	476-661	648-901	725-1042	715-1060	1323-1856	753-1339	381-941
6 mg/kg post-HHD	713-993	965-1342	1078-1552	1064-1563	1165-1719	650-1256	327-885
**7-hour HHD every other day with a total dialysate volume of 30 L (Qd = 4.3 L/hr)**							
4 mg/kg post-HHD	503-704	289-455	663-940	350-619	679-995	361-690	667-996
**6 mg/kg post-HHD**	**760-1059**	**443-683**	**1007-1415**	**536-935**	**1039-1485**	**556-1043**	**1024-1485**
8 mg/kg post-HHD	1008-1411	589-900	1333-1859	714-1240	1376-1970	740-1375	1354-1981
**7-hour HHD every other day with a total dialysate volume of 50 L (Qd = 7.1 L/hr)**							
4 mg/kg post-HHD	498-707	290-445	651-908	346-593	668-945	357-651	679-976
**6 mg/kg post-HHD**	**757-1066**	**436-672**	**984-1367**	**513-898**	**1005-1433**	**526-984**	**1018-1476**
8 mg/kg post-HHD	1006-1419	581-899	1306-1831	687-1188	1336-1902	707-1298	1355-1959
**7-hour HHD every other day with a total dialysate volume of 60 L (Qd = 8.6 L/hr)**							
4 mg/kg post-HHD	502-705	290-448	651-907	341-590	663-942	350-644	671-969
**6 mg/kg post-HHD**	**755-1058**	**435-664**	**976-1350**	**510-878**	**997-1405**	**526-965**	**1009-1446**
8 mg/kg post-HHD	1016-1409	578-890	1304-1810	688-1180	1321-1876	703-1298	1341-1930

Qd: dialysate flow rate; SD: supplemental dose

Shaded boxes indicate the day of a 3-hour HHD session.

Bolded dosing regimens are those with the predicted AUC_{24h} IQRs of 5,000 simulated patients falling within the target AUC_{24h} IQR 465-1422 mg·h/L
